# Formulating a heat- and shear-labile drug in an amorphous solid dispersion: Balancing drug degradation and crystallinity

**DOI:** 10.1016/j.ijpx.2021.100092

**Published:** 2021-07-17

**Authors:** Daniel A. Davis, Dave A. Miller, Supawan Santitewagun, J. Axel Zeitler, Yongchao Su, Robert O. Williams

**Affiliations:** a College of Pharmacy, The University of Texas at Austin, Austin, TX 78712, USA; b DisperSol Technologies, LLC, 111 W. Cooperative Way, Building 3, Suite 300, Georgetown, TX 78626, USA; c Pharmaceutical Sciences, Merck & Co., Inc., Rahway, NJ 07065, USA; d Department of Chemical Engineering and Biotechnology, University of Cambridge, Cambridge CB3 0AS, UK

**Keywords:** Residual crystallinity, Seed crystallinity, Amorphous solid dispersion, Degradation, Bioavailability enhancement, KinetiSol processing

## Abstract

We seek to further addresss the questions posed by Moseson et al. regarding whether any residual crystal level, size, or characteristic is acceptable in an amorphous solid dispersion (ASD) such that its stability, enhanced dissolution, and increased bioavailability are not compromised. To address this highly relevant question, we study an interesting heat- and shear-labile drug in development, LY3009120. To study the effects of residual crystallinity and degradation in ASDs, we prepared three compositionally identical formulations (57–1, 59–4, and 59–5) using the KinetiSol process under various processing conditions to obtain samples with various levels of crystallinity (2.3%, 0.9%, and 0.1%, respectively) and degradation products (0.74%, 1.97%, and 3.12%, respectively). Samples with less than 1% crystallinity were placed on stability, and we observed no measurable change in the drug's crystallinity, dissolution profile or purity in the 59–4 and 59–5 formulations over four months of storage under closed conditions at 25 °C and 60% humidity. For formulations 57–1, 59–4, and 59–5, bioavailability studies in rats reveal a 44-fold, 55-fold, and 62-fold increase in mean AUC, respectively, compared to the physical mixture. This suggests that the presence of some residual crystals after processing can be acceptable and will not change the properties of the ASD over time.

## 1 Introduction

Formulating an amorphous solid dispersion (ASD) with an entirely amorphous formulation (i.e., an ASD with no residual crystals in the composition) decreases the risk of recrystallization during storage and minimizes variation in bioavailability (Hancock, 2002; Lehmkemper et al., 2017; Qian et al., 2010). However, it is difficult to eliminate crystallinity entirely during the processing of an ASD because many processes require shear energy and heat to facilitate the amorphous conversion, which consequently can promote chemical degradation of the drug (Hengsawas Surasarang et al., 2017; Huang et al., 2017; Ma et al., 2019). Therefore, allowing crystallinity decreases the degradation of drugs when complete amorphous conversion produces unacceptable chemical degradation levels (Huang et al., 2017). However, this is a trade-off that has not been extensively reported due to the negative effects associated with the presence of residual crystals in an amorphous composition.

Recently it was reported that residual indomethacin crystallinity (i.e., unconverted, mechanically defected crystals that remain after processing) decreased its amorphous solubility advantage, but it did not promote recrystallization, which suggests that trace amounts of residual crystallinity can have a minimal impact on non-sink dissolution (Moseson et al., 2020). In our study, we seek to explore the relevance of these findings using a drug in development, LY3009120, which exhibits quite challenging physicochemical properties. LY3009120 is heat- and shear-labile, so the drug chemically degrades when using a process that imparts high shear or heat to convert a composition into an ASD. Therefore, the chemical stability of LY3009120, along with its miscibility in the polymer carrier, must be carefully balanced in order to confirm whether the ASD can tolerate some degree of residual crystallinity while still showing acceptable and minimal degradation. Furthermore, the properties of the ASD must not change during storage to negatively affect its long-term stability and bioavailability.

In the amorphous state, the crystalline lattice has been disrupted, creating a high-energy thermodynamic state that decreases the lattice-activation energy to improve the drug's solubility (Hancock and Zografi, 1997). The amorphous state's solubility advantage does not come without consequences, however. For example, the disrupted crystalline lattice's high-energy state is unstable and thermodynamically driven to relaxation, nucleation, and crystallization during storage or during transit through the gastrointestinal tract (GIT) (Hughey and Williams, 2012). In addition to recrystallization, the amorphous state shows (a) less molecular packing than the crystalline state, (b) an increased potential for moisture absorption, and (c) increased molecular mobility. This renders the amorphous state susceptible to increased chemical reactivity and further formulation concerns (Carstensen and Morris, 1993; Xiang and Anderson, 2004). Therefore, it is a primary concern to maintain the physical stability of an ASD to prevent recrystallization and degradation of the drug.

Modulated differential scanning calorimetry (mDSC) and powder X-ray diffraction (pXRD) are two techniques used for studying the recrystallization tendencies of the amorphous phase over time (Dedroog et al., 2020; Hancock and Zografi, 1997). These techniques have several limitations. The pXRD method lacks the sensitivity to detect trace crystallinity in samples. The conventional limit of detection is 5% or lower, depending on the drug (Randall et al., 2010; Thakral et al., 2018). Additionally, the lack of sensitivity of pXRD to distinguish amorphous–amorphous phase separation (Dedroog et al., 2020) and to detect nanocrystallinity (Bates et al., 2006; Einfalt et al., 2013; Pecharsky and Zavalij, 2008) explains why it is often coupled with mDSC analysis (Dedroog et al., 2020). On the other hand, depending on the drug–polymer miscibility, the drug can dissolve into the polymer matrix above the composition's glass transition temperature but below the drug's melting point. This makes the presence of crystallinity undetectable during mDSC analysis (Dedroog et al., 2020). When used together, these two techniques are useful for evaluating many aspects of amorphous samples in the solid state (e.g., crystallinity, miscibility, molecular mobility); however, other analytical techniques are needed to more completely characterize the remaining properties of ASDs (e.g., molecular interactions, particle morphology) (Dedroog et al., 2020).

Terahertz time-domain spectroscopy (THz-TDS) is one emerging technique that can be used to measure molecular interactions. In disordered organic molecular solids, THz-TDS can measure the mobility of molecular dipoles that result from molecular interactions, such as hydrogen bonds or Van der Waals interactions. The degree of these intermolecular interactions can be used as a factor to determine the physical stability of amorphous formulations (Liu et al., 2019). Acid–base ionic interactions are stronger than hydrogen bonds, and it has been shown that drugs with ionic interactions are highly miscible and stable for more than 50 days at 298 K (Yoo et al., 2009).

In addition to measuring molecular mobility, THz-TDS can also be used to detect trace crystallinity in an amorphous formulation. At terahertz frequencies, the crystalline structure produces a unique spectrum of low-energy vibrational modes (e.g., phonon modes, coupled large-amplitude bending, torsion vibrations) when the order is sufficient for coherent motion of the molecules. The absorption spectrum of amorphous materials shows an increasing baseline with no characteristic peaks, while the absorption spectrum for crystalline materials exhibits characteristic peaks. Trace crystallinity can be easily identified with THz-TDS by the presence of peaks detected in the absorption spectrum (Sibik and Zeitler, 2016).

Additionally, mDSC and pXRD are used to calculate the percentage of crystallinity. For example, an intensity–crystallinity calibration curve is constructed by measuring the peak intensity of physical mixtures that have known crystallinity levels to enable determination in the unknown samples (Chen et al., 2001; Thakral et al., 2018). Generally, in vitro dissolution testing is performed under sink conditions, which uses a volume of medium at least three times the volume required to form a saturated solution (2015). Though sink conditions are desirable, they are not mandatory (2015). Non-sink conditions have been adapted for approved ASD products (Sun et al., 2016). The supersaturated state formed in an ASD exhibits different release profiles and recrystallization kinetics that are not reflected when dissolution is performed under sink conditions (Sun et al., 2016; Van Speybroeck et al., 2010). This could mask changes in drug concentration influenced by crystallinity. Therefore, using the sink index to establish non-sink conditions for evaluating ASD performance is suggested in order to reproducibly detect small changes in the dissolution profile caused by residual crystallinity (Sun et al., 2012; Sun et al., 2016).

Recrystallization can occur in the solid state during storage or from the supersaturated state during dissolution. In each case, recrystallization consists of two processes: nucleation and crystal growth. Yoshioka et al. highlight the impact of seed crystals (5% *w*/w) that accelerate crystal growth in the solid state of ASDs (Yoshioka et al., 1994). In this case, the seed crystals act as a nucleation site to accelerate crystal growth under the conditions studied. During dissolution, this translates to a lower maximum achieved concentration of the ASD with increasing levels of crystallinity for water-soluble polymers (Moseson et al., 2020; Ojo et al., 2020). This is important because it is not always possible to eliminate residual crystallinity when processing ASDs. For example, trace crystallinity has been reported in the commercially lyophilized formulation of paclitaxel (Schmitt et al., 2015). Additionally, Trasi et al. showed evidence that a commercially available tacrolimus ASD contains low levels of crystalline tacrolimus as compared to the innovator Prograf® (Trasi et al., 2017). Therefore, systems that accurately model the effects of crystallinity in a formulation are critical.

Previously, crystallinity in ASDs was modeled by spiking a known amount of extraneous, unprocessed, bulk crystalline drug into the formulation (Pack et al., 2017; Rahman et al., 2015; Wegiel et al., 2013). Recently, it has been suggested that better models are needed because the residual seeds after the process can have properties that differ from the bulk crystalline API that was added extraneously (Que et al., 2018). Que. et al. reported that for paclitaxel ASD formulations, up to 18% crystalline content can be added, while producing virtually no effects on dissolution and causing no significant desupersaturation, depending on the crystalline properties present. Desupersaturation is a consequence of secondary nucleation or the growth of seed crystals that reduces the amount of drug in solution available for absorption (Frawley et al., 2012; Ilevbare et al., 2012; Moseson et al., 2020; Que. et al., 2018).

Specifically, Moseson et al. distinguished unique differences in dissolution performance between samples that contained similar amounts of residual crystallinity (i.e., unconverted, mechanically defected crystals that remain after processing, ranging from 1.8–25%) and spiked crystallinity (i.e., entirely amorphous samples spiked with extraneous unprocessed crystalline drug, ranging from 5 to 40% known levels of crystallinity). The residual crystalline samples exhibited slight desupersaturation during the plateau phase when residual crystallinity was higher than 3%, suggesting a degree of crystal seed growth. However, the amorphous sample, spiked with the unprocessed crystalline drug, did not exhibit desupersaturation during the plateau phase (i.e., during crystal seed growth) at any concentration. This desupersaturation of the residual crystalline samples was attributed to the mechanical strain imposed on the crystalline drug during hot-melt extrusion, which generated defects on the surface of the crystal. These surface defects increase the residual crystalline drug's surface area and surface energy, which in turn increases the likelihood of crystal growth during non-sink dissolution. Interestingly, desupersatuartion was not observed when the residual crystalline content fell below 2% (Moseson et al., 2020), suggesting a potential threshold at which the desupersaturation caused by residual crystallinity may be negligible.

Furthermore, Theil et al. showed that among identical amorphous formulations with the same amounts of crystallinity, the origin of the sample's crystallinity determined unique differences in the API's release during dissolution (Theil et al., 2018). In their study, the impact on the dissolution of equivalent amounts of unprocessed crystalline material showed a greater rate and extent of drug release in all cases compared to an equivalent amount of crystallinity that endogenously crystallized during storage (i.e., the amorphous drug recrystallizing due to instability) (Theil et al., 2018). Similarly, Ojo et al. utilized accelerated temperature and humidity conditions to grow controlled amounts of crystallinity (i.e., 5% and 10% crystallinity) in ASDs formulated using a water-soluble polymer (i.e., PVP K12) and a water-insoluble polymer (i.e., HPMCAS) (Ojo et al., 2020). The authors purposely preferred endogenously grown crystals because seed crystals potentially cloud the effects on intrinsic dissolution from the dissimilar nature of endogenously formed and spiked crystallinity. The authors reported the impact of intrinsic crystallinity on the dissolution performance of ASDs for both soluble and insoluble polymers. The authors concluded that with soluble polymers, dissolution performance decreases with increasing crystallinity. However, for insoluble polymers that contain a minor amount of crystallinity (i.e., 5% and 10% crystallinity), the dissolution profile was not affected by the level of crystallinity.

This phenomenon was attributed to the concomitant release of the polymer, amorphous API, and crystalline API, which allows for crystal seed growth in solution, thus giving rise to the desupersaturation phase of soluble polymers. However, in insoluble polymers, the crystals are trapped in the insoluble matrix, which restricts crystal growth to drug diffusion through the matrix. This limits crystal growth and ultimately limits desupersaturation in solution (Ojo et al., 2020). These studies highlight the fact that the presence of crystallinity not only affects dissolution, but changes in the crystalline surface area or surface energy can also negatively affect the dissolution profile and cannot be modeled by spiking with unprocessed crystalline drug (Moseson et al., 2020; Ojo et al., 2020; Que. et al., 2018; Theil et al., 2018). Therefore, once manufactured, large changes (e.g., 5%) in the crystalline content upon storage (irrespective of origin) affects the dissolution profile of ASDs (Frawley et al., 2012; Ilevbare et al., 2012; Que. et al., 2018). However, to our knowledge, the effects of residual crystallinity less than 1% have not been reported.

During stability studies, changes in drug degradant levels and potency are monitored in addition to monitoring the change in dissolution of the drug from the ASD matrix during long-term and accelerated stability conditions (2003). As mentioned above, compared to the crystalline state, the amorphous state's decreased molecular packing increases the potential for moisture absorption, and its higher molecular mobility increases the drug's chemical reactivity (Carstensen and Morris, 1993; Xiang and Anderson, 2004). Consequently, small increases in degradant levels can be detrimental to product development due to ICH thresholds for degradation products in new drug products (ICH Q3B R2 2006). The guidelines for degradation products state the reporting threshold for a dose less than one gram is 0.1%, the identification threshold for a dose of 10 mg to 2 g is 0.2%, and the qualification threshold for a dose of 10 mg to 100 mg is 0.5% (Guidance for industry Q3B(R2), 2006). For example, the generation of any specific degradant in an amount greater than 0.5% requires further toxicity studies to assess the clinically relevant side effects associated with the degradant (Guidance for industry Q3B(R2), 2006; Nguyen et al., 2015).

Considering the nominal amount of degradants allowed in a dosage form, formulation scientists have encountered difficulty in manufacturing ASDs using high-energy and thermal processes, particularly when the drug is heat- and shear-labile or when the drug undergoes excipient-induced degradation in these circumstances (Dong and Choi, 2008; Hengsawas Surasarang et al., 2017; Monschke et al., 2020; Tan et al., 2020). In certain situations, especially for heat- and shear-labile compounds, eliminating trace crystallinity requires a higher energy input (Thompson and Williams, 2021), but crystallinity is eliminated at the expense of increasing degradant levels. In the present study, we investigate the processing parameters that create ASDs that contain specific trace amounts of residual crystallinity as a way to control the level of degradants and the resulting impact this residual crystallinity has on stability, dissolution, and bioavailability.

**Fig. 1:**
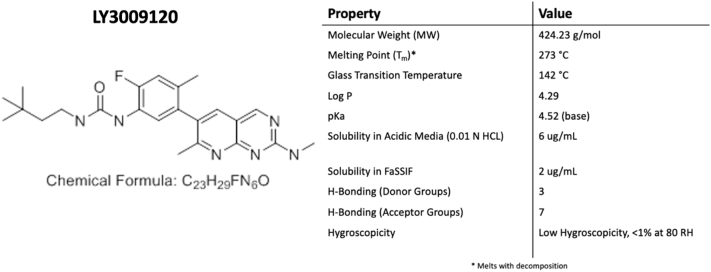
Chemical structure and relevant properties of LY3009120 (Wesley, 2017).

To accomplish this, LY3009120, a drug in development, was used because it represents a highly challenging drug to formulate into an ASD because (a) it is heat- and shear-labile, (b) it has low solubility in organic and aqueous solvents, and (c) it has a high melting point (> 200 °C). Fig. 1 shows the structure and relevant physicochemical properties of LY3009120 (Wesley, 2017). In this study, we evaluated the impact of trace amounts of residual crystallinity after KinetiSol processing on the long-term stability, dissolution performance, and bioavailability of the drug in order to determine whether allowing residual crystallinity for heat- and shear-labile compounds is an effective approach to generate a stable ASD while minimizing degradation.

## 2 Materials and methods

### 2.1 Materials

Candurin was purchased from EMD Performance Materials (Philadelphia, PA). Copovidone (Va64), a water-soluble copolymer of vinylpyrrolidone and vinyl acetate, was purchased from BASF (Ludwigshafen, Germany). Sodium stearyl fumarate was purchased from JRS Pharma LP (Patterson, NY). HPLC grade methanol, acetonitrile, and trifluoroacetic acid (TFA) were purchased from Fisher Scientific (Pittsburgh, PA). Fasted-state simulated intestinal fluid (FaSSIF) was purchased from Biorelevant.com Ltd. (Surrey, United Kingdom). Sodium chloride, potassium salt, and 1 M HCL were purchased from Fisher Scientific. All other reagents used were of ACS grade or higher.

#### 2.1.1 LY3009120

Lilly and Co. (Indianapolis, IN) donated the drug, LY3009120. Studies were based on a 50 mg dose of LY3009120, and the qualification threshold for any degradant was 0.5%. The major degradant of LY3009120, Degradant A, was previously proposed to be a result of hydrolysis (Wesley, 2017). An increase in shear stress directly increased the amount of Degradant A. Therefore, Degradant A was used as a surrogate marker to follow the impact of shear stress on the degradation of LY3009120. Other degradants detected were below the identification threshold. The reported value of the degradant's peak is a percentage of its area relative to the parent LY3009120 peak observed during HPLC analysis. Various processing conditions were selected to manufacture LY3009120 formulations that showed different degradation levels and crystallinity, as described further below.

### 2.2 Evaluating the effects of crystalline LY3009120 on stability and bioavailability

#### 2.2.1 KinetiSol processing and milling

The KinetiSol processing research formulator, KBC20, was used to process samples (DisperSol Technologies, LLC, Georgetown, TX). Physical mixtures of LY3009120:SSF:Candurin:Va64 (10:4:3:83) were prepared by hand-mixing in plastic bags for 5 min. Candurin is used in the composition as a processing aid, and has previously been shown to not interact with the API (Davis et al., 2020). The ejection criteria for KinetiSol processed samples are based either on the processing temperature or the time. Both are used in this study. The temperature of the composition is monitored in real time using a fiber optic IR probe. During optimization, the batch size ranged from 10 to 15 g. Based on the results, 15 g was chosen as the optimal amount. Samples were manually quenched between metal plates with and without liquid nitrogen, depending on the sample. When liquid nitrogen was used, it was poured over the metal plates before starting the formulator. The formulator was activated after the liquid nitrogen evaporated, and the ejected samples were quenched on the cooled plates. Immediately after quenching, all samples were milled using an IKA tube mill control (IKA-Werke, Staufen, Germany) operated at 20,000 rpm for 30 s with a 15-s pulse. All formulations studied, 57–1, 59–4 and 59–5, are composed of LY3009120:SSF:Candurin:Va64 (10:4:3:83).

#### 2.2.2 Stability study

Stability studies were performed to monitor changes in crystallinity and purity over time. Accelerated and long-term conditions were used for evaluation. The long-term conditions were 25 °C and 60% relative humidity, while the accelerated conditions were 40 °C and 75% relative humidity. Two formulations were placed on stability, LY3–59-4 and LY3–59-5, ssNMR studies were only conducted on samples stored at 25 °C and 60% relative humidity. Four grams of both formulations were exposed to the long-term and accelerated conditions in both the open and closed state. For the closed samples, a 1 g silica gel desiccant canister was placed inside a 60 cc HDPE bottle with the formulation and sealed with the secuRX ribbed side white closure. For the open conditions, a permeable membrane was used to expose the sample to the chamber's relative humidity while preventing contamination. The purity time points were taken at time zero, at four months, and at the conclusion of the study. Crystal growth was monitored at 2, 8, 16, and 24 weeks.

#### 2.2.3 High-pressure liquid chromatography

Sample purity was analyzed using a Thermo Scientific Dionex UltiMate 3000 high-pressure liquid chromatography (HPLC) system (Thermo Scientific, Sunnyvale, CA) with Chromeleon 7 software for data acquisition and analysis. The following HPLC conditions were used for separation: a column temperature of 30 °C, a flow rate of 1.5 mL/min, an injection volume of 2 μL, and a detection wavelength of 239 nm. Mobile phase A was composed of 0.1% TFA in water, and mobile phase B was composed of 0.1% TFA in acetonitrile. For separation, a Zobrax Bonus-RP column with a 4.6 mm internal diameter, 75 mm length, and a 3.5 μm particle size was used (Serial Number: USTM002935).

During analysis, a gradient extended from 0 min to 9.5 min (95% A ➔ 23% A). From 9.5 min to 12.1 min, the system was held isocratic (23% A, 77% B). A second gradient was extended from 12.1 min to 13 min (23% A ➔ 5% A). The system was held isocratic from 13 min to 16 min (5% A, 95% B). A third gradient was extended from 16 min to 16.1 min (5% A ➔ 95%). Last, the system was held isocratic from 16.1 min to 20 min (95% A, 5% B). The total run time for the method is 20 min. LY3009120 has a retention time of 5.9 min, and degradants A, B, and C have a relative retention time of 0.49, 0.55, and 0.85, respectively. Samples were prepared using 80:20 methanol: water as a diluent, sonicating for 5 min. Last, the samples were filtered through a 0.22 μm PTFE filter. A target concentration range of 0.2–0.3 mg/mL was used during sample preparation, as linearity was determined within this range. The potency of the processed formulations was above 85%.

#### 2.2.4 Non-sink pH-shift microdissolution

The dissolution of the LY3009120 formulations was performed using a Pion MicroDISS Profiler (Pion Inc., Boston, MA) to determine the different formulation's release rates and solubility concentrations. The apparatus utilized a 20 mL dissolution vessel, which maintained the dissolution media at 37 °C ± 0.2 °C by circulating water through a Julabo Corio CD immersion circulator (Julabo USA, Inc., Allentown, PA). Based on safety data from the clinical trial of LY3009120, 50 mg LY3009120 doses were selected for this experiment (2019). To maintain a dose of LY3009120 relevant for the microdissolution volume, 1.11 mg of LY3009120 (an 11.11 mg formulation) was used in 20 mL. This maintains a constant concentration if the dissolution were to be performed in 900 mL.

After the first collected spectra at *t* = 0 min, 12 mL of 0.01 N HCL was added to the dissolution vessel. At *t* = 30 min, a pH shift was performed by adding 8 mL FaSSIF in pH 6.8 phosphate buffer. The dissolution was performed for 6 h while stirring at 150 rpm with cross stir bars. LY3009120 concentrations were measured every 30 s for the duration of the study using UV probes (with a 5 mm path length) between the wavelengths of 300 nm and 310 nm in the acidic phase and 295 nm and 305 nm in the basic phase via a Rainbow UV spectrometer (Pion Inc., Boston, MA).

#### 2.2.5 In VIVO pharmacokinetic study in SD rats

A pharmacokinetic study was conducted under an animal protocol approved by Pharmaron, Inc. (Beijing, China), following the recommendations outlined in the Guide for the Care and Use of Laboratory Animals of the National Institutes of Health (Animal Use Protocol # PK-R-06012020). The pharmacokinetic parameters of LY3009120 were studied on Sprague Dawley (SD) rats after oral (PO) administration. These 20 rats were divided into four groups (*n* = 5). The animals were fasted overnight before dosing.

The concentration of LY3009120 in the formulation dosed was adjusted for potency to 2.85 mg/mL LY3009120 for all formulations. The formulation preparation procedure was as follows: (1) While stirring at 350 rpm, the allocated amount of the formulation is dropped into 20 mL of 0.01 N HCl. (2) After 3 min, 16 mL of 0.01 N HCL is added and stirred for an additional 5 min. (3) Immediately after stirring, the five rats in the group were dosed using oral gavage (dose level 2.85 mg/kg) within 5–10 min after mixing. Before dosing each animal, the syringe was washed with 0.5 mL of 0.01 N HCL. These steps were then repeated for each group.

Blood samples (200 μL) were collected from the jugular vein, centrifuged at 4000 *g* for 5 min in a 4 °C centrifuge, then stored in a freezer at −75 °C before analysis. Blood samples were taken from each animal at 0.25, 0.5, 1, 2, 4, 6, 8, and 24 h. Plasma was analyzed using an LC-MS/MS method to determine LY3009120 content, and the respective pharmacokinetic parameters were determined using Phoenix WinNolin v6.1 (Certara, Princeton, NJ).

### 2.3 Solid-state characterization

#### 2.3.1 Powder X-ray diffraction

The crystalline content was determined using powder X-ray diffraction (pXRD) using a MiniFlex 600 (Rigaku Americas Corporation, The Woodlands, TX). The instrument utilized a Cu–Kα radiation source generated at 40 kV and 15 mA. Powder samples were loaded into an aluminum sample holder and leveled using a glass side before placing them in the sampler. Instrument parameters scanned a two-theta range of 5.0–35.0° with a step size of 0.02° and a scan speed of 5.0°/min while rotating the sample. A calibration curve using known amounts of crystallinity (i.e., 1%, 2%, 4%, and 8% crystalline LY3009120) was constructed to calculate the crystalline content in the formulations. The data were processed using PDXL2 software (Rigaku Corporation, Tokyo, Japan).

The pXRD analysis of stability samples produced high intrasample variability, suggesting changes in crystallinity (see Table 1). However, upon visual inspection of the overlays in Fig. S1, no change in the crystallinity of LY3009120 is suggested. This suggests that pXRD is unable to differentiate between small changes in crystallinity at low crystalline content. It also suggests that a more sensitive technique is needed to confidently quantitate crystallinity for this study (e.g., ssNMR).

**Table 1:** Summary of the percentage of crystallinity of 59–4 and 59–5 formulations under all conditions across all time points. The intensity of LY3009120's Bragg's peak at 7.1° was used for pXRD crystallinity analysis.

Time Point	59–4 (% Crystallinity)				59–5 (% Crystallinity)			
	25/60 Closed	25/60 Open	40/75 Closed	40/75 Open	25/60 Closed	25/60 Open	40/75 Closed	40/75 Open
T_O_	1.02				0.38			
2 Weeks	0.60	0.58	0.78	0.42	0.18	0.16	0.22	0.25
2 Months	1.27	0.70	0.85	1.98	0.35	0.36	0.45	0.40
4 Months	0.54	0.58	0.33	0.86	0.12	0.30	0.21	0.46
Post-stability dissolution	0.83				0.50			

#### 2.3.2 Terahertz time-domain spectroscopy

Intermolecular interactions were measured using terahertz time-domain spectroscopy (THz-TDS). Samples were gently dispersed into fine particles using a mortar and pestle. Polyethylene (PE) powder was added to the samples with a sample:PE ratio of 1:9, then mixed using an agate mortar and pestle by geometric mixing. PE is commonly used as a binder and diluent in THz measurements because PE is nearly transparent in the terahertz region [B].

The sample powder was compressed under a 2-ton load in a 13 mm flat-faced pellet die for 1 min using a hydraulic press. Three pellets were prepared for each formulation. Pure PE pellets were prepared for use as the reference for the terahertz measurements. The transmission terahertz measurements were performed at room temperature using a commercial Terapulse 4000 spectrometer (TeraView Ltd., Cambridge, UK). Time-domain waveforms of 50 ps duration were recorded over a co-averaging time of 6.67 s, and the absorption spectra in the transmission were calculated following the Fourier transformation of the sample and reference waveforms. A constant flow of dry nitrogen gas was used to purge the sample compartment throughout the terahertz measurement to remove any residual water vapor. In order to avoid any artifacts due to potentially inhomogenous mixing, each pellet was measured at four locations to determine the variability in each pellet. The terahertz absorption spectra were obtained in the frequency range of 0.3–2.5 THz.

#### 2.3.3 Solid-state nuclear magnetic resonance

We utilized ^19^F ssNMR to quantify the crystalline content of LY3009120 in quaternary ASD compositions (Correa-Soto et al., 2017; Huang et al., 2018; Li et al., 2021). All data were acquired on a wide-bore Bruker Advance III HD spectrometer operating at 400 MHz ^1^H Larmor frequency in Biopharmaceutical NMR Laboratory (BNL) (Pharmaceutical Sciences, Merck & Co., Inc. West Point, PA 19486). Powder samples of approximately 90 mg each were packed into 4.0 mm ZrO_2_ rotors. A triple-resonance ^1^H/^19^F/X Magic Angle Spinning (MAS) probe tuned to ^1^H and ^19^F modes was used for all experiments. ^1^H—^19^F cross polarization (CP) MAS spectra were acquired at the Hartmann–Hahn match at a 50 kHz field strength at 294 K, and a MAS frequency of 12 kHz. A 71 kHz TPPM ^1^H decoupling was applied during acquisition. ^1^H and ^19^F T_1_ relaxation times were measured using the saturation–inversion–recovery method and fitted to be approximately 1.0 s and 30.1 s, respectively (data not shown). The ^19^F-detected cross-depolarization using a recycle delay of 6.0 s, optimized to allow for full ^1^H relaxation of the crystalline drug.^19^F chemical shifts were referenced to the ^19^F peak of teflon at −122 ppm.

## 3 Results

### 3.1 The impact of residual crystallinity on stability using ssNMR quantification

KinetiSol processing was used to create LY3009120 ASDs containing various levels of residual crystallinity and degradation. Table 2 shows the calculated percentage of the compositions' crystallinity, the total degradation from processing, and the degradation product (Degradant A).

**Table 2:** The % crystallinity of LY3009120 in the formulation, total degradation, process-related degradants, and the percentage of the single largest degradant present.

Sample name	% Crystallinity (ssNMR quantitation)	Total Degradation (% AUC)	Degradant A^a^ (% AUC)
Physical Mixture	9.2	0	0
57–1	2.3	0.74	0.12
59–4	0.9	1.97	0.68
59–5	0.1	3.12	1.14

a Degradant A is used as a surrogate marker to follow the impact of shear stress on the degradation of LY3009120.

Sample 59–5 exhibited the lowest crystallinity levels and the highest level of degradation for an amorphous sample. Sample 59–4 showed less degradation but increased crystalline LY3009120. Last, Sample 57–1 is an example of a product with acceptable degradation levels at the expense of a significantly higher level of crystallinity. Of these, Samples 59–4 and 59–5 were placed on stability.

Long-term and accelerated stability conditions were utilized to assess crystal growth, which results in a change in drug release and bioavailability. Samples 59–4 and 59–5 were evaluated in the open and closed state under two conditions: (a) 25 °C and 60% relative humidity (25 °C/60% RH) and (b) 40 °C and 75% relative humidity (40 °C/75% RH) over four months. Due to the high melting point of LY3009120 (i.e., 273 °C), degradation of the polymer (i.e., copovidone) begins before the melting point of LY3009120 is reached. This complicates the mDSC analysis and prevents clear identification of the melting event (see Fig. S2). However, before degradation, a single T_g_ was observed at 109 °C, indicating a single-phase system with LY3009120 molecularly dispersed in the formulation (Qian et al., 2010).

ssNMR (as an inherently quantitative method) was used to measure the low levels of crystallinity present in the samples. mDSC cannot calculate the percentage of LY3009120 crystallinity, and pXRD measurements lack sensitivity when measuring low levels of crystallinity (see Fig. S1) (Li et al., 2020). ^19^F ssNMR quantification improves measurement sensitivity by 3-fold compared to ^13^C quantification. This allows ^19^F ssNMR to quantify crystalline content less than 0.5% *w*/w (Asada et al., 2016; Li et al., 2020).

**Fig. 2:**
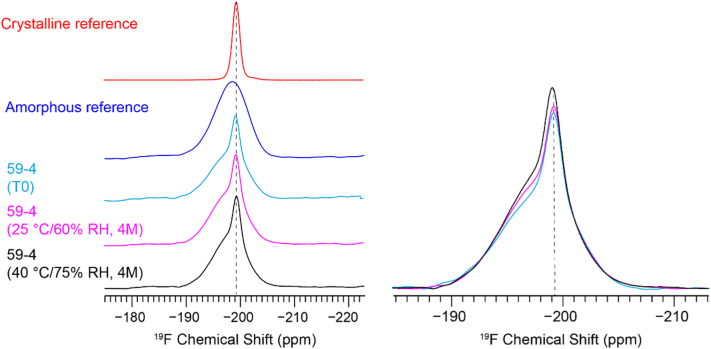
^19^F ssNMR quantification of crystallinity in LY3009120 ASDs. Left: an array of ^19^F CP-MAS spectra of crystalline and amorphous references, 59–4 ASDs at T_0_, 25 °C/60% RH (4-month) and at 40 °C and 75% RH (4-month). Right: Overlaid spectra to illustrate the various crystalline contents. A reference-based method was utilized for 19F quantification (Huang et al., 2018).

A reference-based method was developed and utilized in this study (Huang et al., 2018). The fluorine present in the molecule offers an excellent sensitivity for its high gyromagnetic ratio and natural abundance. This generates peaks that can be reliably integrated and differentiated, as seen from the intense peak associated with crystalline LY3009120 in Samples 59–4 (at T_0_) and 59–4 (at 25 °C/60% RH) and in Sample 59–4 (at 40 °C/75% RH). These peaks can distinguish between 0.9%, 1.0%, and 1.2% crystallinity in LY3009120, respectively (see Fig. 2). The spectral comparison suggests that ^19^F ssNMR quantification was sensitive enough to differentiate between samples down to a 0.1% difference in crystallinity.

The ^19^F ssNMR technique determined the exact crystalline content at time zero for Samples 57–1, 59–4, 59–5, and in the physical mixture, along with the 4-month time point for both 59–4 and 59–5 in the closed state under 25 °C/60% RH and 40 °C/75% RH conditions. The crystallinity content is reported as a percentage of the crystalline drug with respect to either the total formulation or in relation to the total drug content (see Table 3). The percentage crystallinity of the total drug is calculated by dividing the amount of crystalline drug detected by the total drug content in the sample. Reporting crystallinity in this manner gives insight into the amorphous fraction of the drug in each formulation.

**Table 3:** The percentage crystallinity relative to the total formulation and relative to the total API content. Values were determined using ssNMR analysis. “Closed” refers to the stability samples stored in a closed container with desiccant.

	Time Zero (T_0_)				4-Month 25 °C/60 RH Closed		4-Month 40 °C/75 RH Closed	
	PM	57–1	59–4	59–5	59–4	59–5	59–4	59–5
% Crystallinity of total formulation	9.2%	2.3%	0.9%	0.1%	1.0%	0.2%	1.2%	0.3%
% Crystallinity of total drug	100%	26.9%	10.8%	0.9%	12.0%	2.9%	14.2%	3.5%

The presence of residual crystallinity after KinetiSol processing did not promote crystal growth in samples after four months on stability under closed conditions at either 25 °C/60% RH or 40 °C/75% RH. This indicates a stable ASD. LY3009120 contains enriched C=O and C—F groups. Fluorinated drugs (e.g., posaconazole) have been found to form C=O···H–O and O–H···F hydogen bonds with polymers (Lu et al., 2019; Lu et al., 2020). As a hypothesis, the drug–polymer interactions in LY3009120 ASDs likely contribute to this stability.

**Fig. 3:**
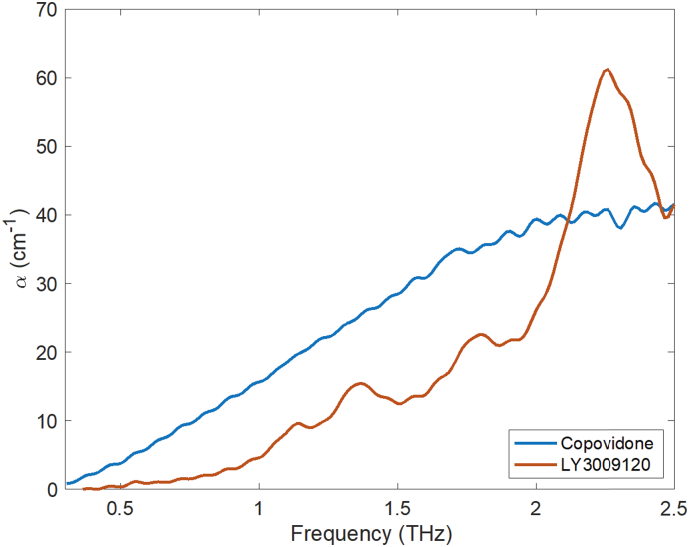
The absorption coefficient of copovidone and LY3009120 as measured using THz-TDS.

The presence of drug–polymer interactions plays an important role in achieving such stability. The degree of interaction can be measured based on the absorption coefficient values obtained from the terahertz measurements. Fig. 3 shows the absorption spectra of copovidone and the crystalline form of LY3009120. Since copovidone is highly amorphous, its absorption spectrum shows an increasing spectral baseline with no distinct peaks, as is expected for any amorphous solid.

**Fig. 4:**
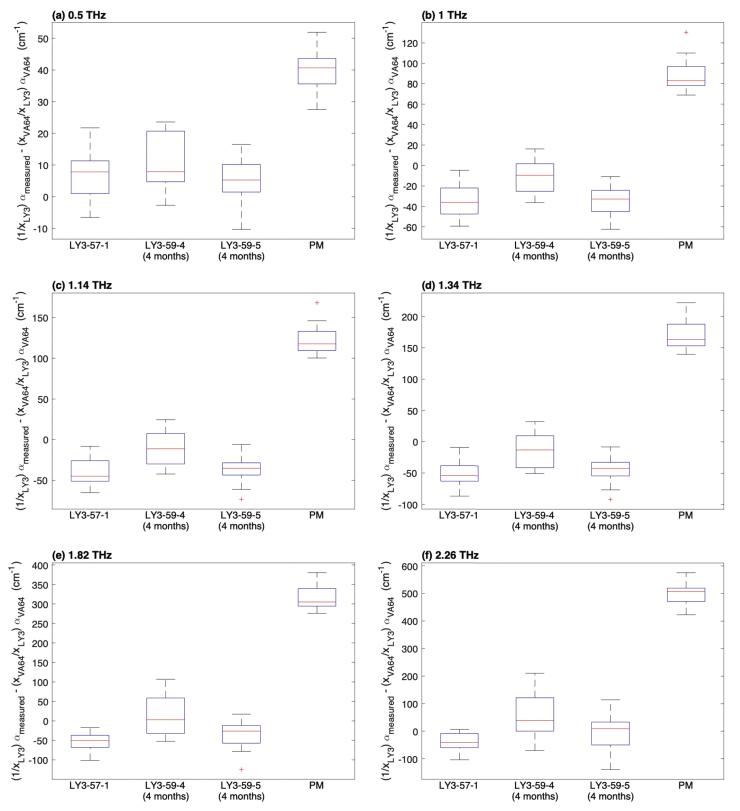
The absorption coefficient of LY3009120 in KinetiSol formulations and in the physical mixture at selected frequencies of 0.5, 1, 1.14, 1.34, 1.82, and 2.26 THz relative to the amount of copovidone present. *X* represents the mass fraction.

In contrast, the spectrum of the crystalline form of LY3009120 exhibits multiple distinct peaks at frequencies of 1.14, 1.34, 1.82, and 2.26 THz. For the formulations 57–1, 59–4, 59–5, as well as the physical mixture, the absorption at these frequencies is plotted normalized to the absorption due to the given amount of copovidone in these formulations, as shown in Fig. A2. (No absorption is observed at terahertz frequencies in the other excipients due to the lack of mobile dipoles.) In addition to the frequencies at which crystalline vibrational modes are expected, the frequencies of 0.5 THz and 1 THz were selected for additional plots to probe the response of the vibrational density of states (VDOS) in the disordered phase (Sibik et al., 2015). From Fig. 4, it can be seen that the absorption coefficient for all KinetiSol formulations (57–1, 59–4, 59–5) was significantly lower than the physical mixture. This suggests that KinetiSol processing caused intermolecular interactions.

This reduction in the absorption coefficient was observed not only for the frequencies that correspond to the crystalline absorption features but also for the frequencies that were selected to probe the VDOS. Therefore, it can be concluded that absorption is decreased due to (a) interactions between the drug and the polymer matrix through hydrogen bonding and (b) the subsequent decrease in net molecular mobility in the ASD compared to the physical mixture. The low absorption coefficient can therefore be considered to indicate strong drug–polymer interaction and less molecular mobility. This may in turn be indicative of higher physical stability. As expected, the measurements of the physical mixture resulted in a much higher absorption coefficient due to the absence of intermolecular interactions.

In addition to detecting crystal growth during stability, the reported crystallinity values are used to examine the impact of various amounts of residual crystallinity on the dissolution and bioavailability of the drug. Under all conditions, sample purity did not change at any time point.

### 3.2 The impact of residual crystallinity on drug release and supersaturation during dissolution testing

**Fig. 5:**
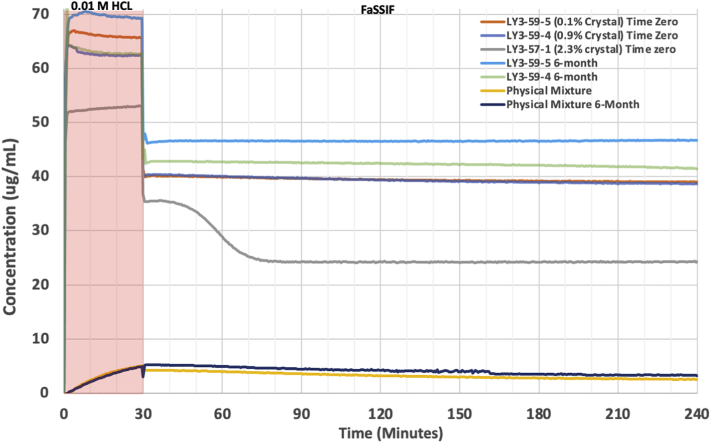
Results from the non-sink, pH-shift microdissolution testing to assess the impact of various degrees of crystallinity on drug release and sustained supersaturation for samples at time zero and after stability storage at 25 °C/60% RH.

Dissolution testing was performed to determine whether the amount of residual crystallinity affected drug release or promoted desupersaturation for formulations 57–1, 59–4, 59–5, as well as the physical mixture. At time zero, residual crystallinity less than 1% (i.e., 59–4 and 59–5) did not affect the release of LY3009120 or promote desupersaturation. However, when the residual crystallinity was greater than 1% (i.e., 57–1), a loss in solubility advantage was observed in the acidic phase. This was attributed to the decreased amorphous fraction of the drug available. Desupersaturation occurred shortly after pH transition (see Fig. 5), indicating a threshold of residual crystallinity that is tolerable before desupersaturation occurs. After the stability study, the samples that contained residual crystallinity less than 1% (i.e., 59–4 closed 25 °C/ 60% RH and 59–5 closed 25 °C/ 60% RH) maintained their solubility advantage and did not undergo desupersaturation.

### 3.3 Evaluating the effects of crystalline LY3009120 on bioavailability

**Fig. 6:**
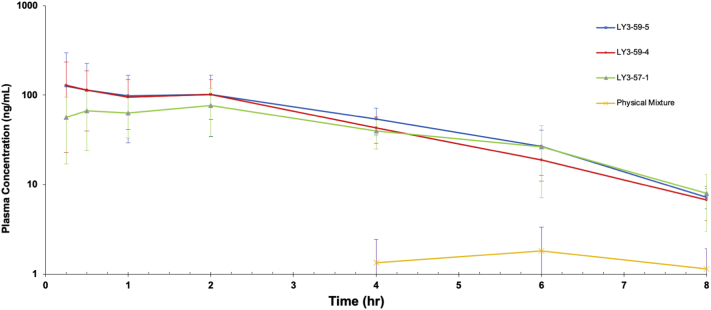
Mean plasma concentration of LY3009120 as a function of time after 28.5 mg/kg oral administration in SD rats. LY3009120 concentrations were undetectable at 24 h in all samples.

Despite formulation 57–1 experiencing desupersaturation during dissolution testing, all processed formulations exhibited increased solubility enhancement in the acidic and neutral phases during dissolution testing, as compared to the corresponding physical mixture. Therefore, an in vivo study in SD rats was conducted to evaluate how various levels of residual crystallinity affect the bioavailability enhancement of Samples 57–1, 59–4 (stored for 4 months in a closed container at 25 °C/60% Rh), and 59–5 (stored for 4 months in a closed container at 25 °C/60% RH). After oral administration, LY3009120 plasma concentrations were measured (see Fig. 6). Each of the KinetiSol processed samples (57–1, 59–4, and 59–5) exhibited measurable plasma concentrations at 0.5 h. However, the physical mixture exhibited no measurable LY3009120 plasma concentrations until 4 h. At the 24 h time point, LY3009120 plasma concentrations were undetectable in all formulations.

Relevant pharmacokinetic parameters were calculated using the plasma concentrations of LY3009120 (see Table 4). Statistically significant increases in AUC_0-Last_ compared to the physical mixture are reported for all processed samples: 59–5, 59–4, and 57–1, with *p*-values of 0.005, 0.003, and 0.0006, respectively. The relative bioavailability enhancement (i.e., the F value) is calculated by dividing the AUC_0-Last_ of the processed sample by the AUC_0-Last_ of the physical mixture. No dose adjustment was required, since all animals received equivalent amounts of LY3009120. The F-value for Samples 59–5, 59–4, and 57–1 were 61.9×, 54.9×, and 43.9× higher than the physical mixture, respectively. Statistical significance was not established between the processed samples, despite the decreased concentrations observed in Sample 57–1 during in vitro dissolution. Due to the variability in blood concentrations among the SD rats, a larger sample size would be needed to determine a statistical significance of bioavailability enhancement in the processed samples.

**Table 4:** Comparison of (a) the relevant pharmacokinetic values for the LY3009120 ASD formulations to (b) the crystalline physical mixture reference.

		Physical Mixture		59–5		59–4		57–1	
PK Parameters	Units	Average	% CV	Average	% CV	Average	% CV	Average	% CV
C_max_	Ng/ml	1.83	83.2	154	158	144	67.9	90	46.6
T_max_	Hr	6.40	14	1.95	97	0.65	117	1.55	98.4
T _½_	Hr	N/A	N/A	2.55	87.1	1.50	14	1.32	8.1
AUC_0-Last_	Ng^a^hr/mL	7.8	5.9	483	191	428	151	342	79
F Value^a^	Unitless	1		61.9		54.9		43.9	

a F value is calculated using the average AUC_0-Last_ of the formulation divided by the physical mixture.

## 4 Discussion

### 4.1 ssNMR quantitation confirms that residual crystallinity did not affect ASD stability

Recently, it has been suggested that residual crystallinity, if sufficiently stabilized by the polymeric carrier, can have a minimal impact on non-sink dissolution. However, generating ASDs with trace amounts of residual crystallinity is not beneficial if these ASDs do not maintain stability during storage or fail to enhance bioavailability. Therefore, in addition to dissolution testing, we evaluated the impact of trace amounts of residual crystallinity on stability and bioavailability.

ssNMR quantitation was used to monitor small changes in LY3009120 crystallinity between formulations during stability. ssNMR is used frequently to characterize the properties of drugs, including polymorphism, phase conversion, quantification (e.g., amorphous drug content), miscibility, homogeneity, drug–polymer interaction, and structural investigation (Li et al., 2020). Previously, Asada et al. reported that ssNMR quantification can detect 0.5% *w*/w crystalline atorvastatin in a formulation (Asada et al., 2016).

In the present study, 0.1% w/w crystalline LY3009120 amounts were detected at time zero in formulation 59–5 (see Table 3). ^19^F has a 4-fold higher gyromagnetic ratio and a 100-fold higher natural abundance than ^13^C (Li et al., 2020). In addtion, fluorine is mostly absent in pharmaceutical excipients (Sheskey and Owen, 2006). As seen in Fig. 2, all the samples are quaternary compositions, but fluorine is present only in LY3009120. This eliminates the need to deconvolute overlapping peaks, and it ultimately simplifies analysis and increases sensitivity by integrating a single peak. This high sensitivity to detect 0.1% w/w differences in crystallinity provides confidence when differentiating crystallinity between samples and during stability. The residual crystallinity present in formulations 59–4 and 59–5 (i.e., 0.9% and 0.1%, respectively) did not promote crystal growth during stability, thus confirming the presence of a stable ASD despite trace amounts of residual crystallinity. It has been reported that introducing spiked crystallinity into an amorphous sample increases the rate of crystal growth during stability (Yoshioka et al., 1994); however, the higher crystallinity in formulation 59–4 compared to 59–5 did not predispose the ASD to recrystallization.

The formulations' stability is attributed to their high T_g_, strong drug–polymer interactions, and a low thermodynamic driving force (i.e., low drug loading). Nucleation and recrystallization of an amorphous drug is a dynamic process that is dependent on several factors: molecular mobility, environmental stress, thermodynamic properties (i.e., enthalpy, entropy, and Gibbs free energy), and preparation method (Baghel et al., 2016). Furthermore, formulation stability increases with increasing T_g_ values (Li et al., 2015). This is partly due to molecular mobility being a driving force for recrystallization (Nunes et al., 2014). The lower the molecular mobility, the higher the degree of intermolecular interactions and the more stable the formulation (Sibik et al., 2015).

The formulation has a high T_g_ (i.e., 107 °C) because the KinetiSol process does not require plasticizers, which are known to decrease the system's T_g_ (DiNunzio et al., 2010). Recrystallization rates increase above the formulation's T_g_; therefore, limiting molecular mobility promotes stability (Nunes et al., 2014). Additionally, Keratichewanun et al. reported that for homogenous samples (e.g., 59–4 and 59–5), the crystallization tendency is inversely proportional to the degree of drug–polymer interactions (Keratichewanun et al., 2015). Therefore, it is suggested that the formulations' stability can be attributed to strong drug–polymer interactions between LY3009120 and copovidone. This fact is of particular importance because it has been recently established that the calorimetric glass transition temperature alone is not a very reliable indicator of physical stability against recrystallization, given the prominent role of sub *T*_*g*_ mobility (Kissi et al., 2018; Ruggiero et al., 2017).

The degree of drug–polymer interactions can be measured at terahertz frequencies, and there is a direct proportionality between the absorption coefficient and molecular mobility in systems with infrared active dipoles. Increased molecular mobility results in increased absorption (Zeitler et al., 2007). If multiple components are present in a mixture, and if no interactions occur between the components, the THz absorption spectrum of the mixture will show the sum of THz absorption spectra of the pure components. However, if the components do interact with each other, the absorption spectrum of the mixture is decreased as the hydrogen bonds of the pure components are disrupted.

Previously, Mensink et al. measured the absorption coefficients of pure protein, pure sugar, and a protein–sugar mixture to study intermolecular interactions. Sugars of various sizes (i.e., trehalose, inulin, and dextran) were used in the study to determine the effect of the molecular size of the sugar on the intermolecular interactions. Trehalose, the smallest sugar used, showed the strongest interactions with the protein (BSA), and the absorption coefficient of trehalose–BSA is lower than that of trehalose or BSA alone. The absorption coefficient of inulin–BSA fell between the absorption coefficients of pure inulin and pure BSA, which indicates fewer interactions between inulin and BSA in the mixture (Mensink et al., 2015).

In the present study, all KinetiSol formulations are shown to have lower absorption coefficients than the physical mixture (see Fig. 4). This indicates that KinetiSol processing formed molecular interactions during processing, which stabilized the formulation in the presence of residual crystallinity. Furthermore, the degree of supersaturation is a driving force of nucleation and recrystallization (Rodríguez-Hornedo et al., 2006; Zhou et al., 2008). However, when drug loading is within the polymer's miscibility limits, the thermodynamic driving force towards recrystallization is reduced (Keratichewanun et al., 2015). The miscibility of the drug in a polymeric carrier is affected by many factors, some of which are molecular mobility and drug–polymer interactions.

These drug–polymer interactions can inhibit crystallization. It has been shown that the level of crystallinity is inversely proportional to the miscibility level (Yoo et al., 2009). Polymers are typically used in the formulations of amorphous drugs in order to stabilize the formulation by lowering its molecular mobility. Mistry et al. reported that using low concentrations of polymer in the drug–polymer system reduced the molecular mobility of the formulation compared to the pure drug. As the polymer concentration used in the formulation increases, the molecular mobility further decreases. This in turn increases the physical stability of the formulation and raises the temperature at which crystallization occurs (Mistry et al., 2015). The THz data have shown that KinetiSol formulations result in a miscible system because the KinetiSol formulations have a lower absorption coefficient than the physical mixture. We find no evidence for bulk phase separation in the terahertz analysis. The low relative absorption coefficient indicates reduced molecular mobility and high drug–polymer interactions, thus it also indicates the potential for high physical stability.

Last, the authors speculate that during storage, there is a maximum crystallinity threshold in primarily amorphous formulations before recrystallization (similar to what is seen during dissolution). In this study, the residual crystallinity in the formulation is so minimal (e.g., 0.1% for Sample 59–5), the residual crystalline particles in the formulation may not act as seeds for nucleation because they are protected by the polymer and separated spatially from other drug particles. Preventing recrystallization in an ASD when residual crystallinity is present would require a homogenous system, a high T_g_, reduced thermodynamic driving forces (i.e., within the drug–polymer miscibility limit), and drug–polymer interactions.

The high sensitivity and reproducibility of ssNMR quantification (Byard et al., 2005; Lefort et al., 2004; Li et al., 2020; Wabuyele et al., 2017) allows for differentiation between the small differences in residual crystallinity in Samples 59–5, 59–4, and 57–1. pXRD analysis could not detect these differences. Therefore, we can use ssNMR to study the impact of residual crystallinity on formulation stability, dissolution performance, and bioavailability enhancement.

### 4.2 Dissolution testing confirms a threshold for desupersaturation in samples with residual crystallinity

Dissolution testing was performed on formulations containing various amounts of residual crystallinity up to a critical threshold at which residual crystallinity negatively affects dissolution. Dissolution testing of samples at time zero determined that when residual crystallinity was below 1% (i.e., formulations 59–4 and 59–5), changes in the extent of drug release or desupersaturation did not occur during the 4 h sampling period. However, when the residual crystallinity was above 2% (i.e., formulation 57–1), drug release decreased, and desupersaturation occurred shortly after the pH transition (see Fig. 5).

Previous studies support these results. Mosseson et al. determined that when residual crystallinity was below 2% (e.g., 1.9% or 1.8%), the residual crystalline samples were indistinguishable from an ASD that had no residual crystallinity. Residual crystallinity above 3% resulted in desupersaturation. Similarly, Ojo et al. reported that when 5% residual crystallinity was present in a water-soluble polymer, the residual crystallinity resulted in desupersaturation.

The ability of ssNMR quantitation to detect trace amounts of residual crystallinity (i.e., 0.1%) allows for a confident conclusion that, for our system, residual crystallinity below 1% did not negatively affect dissolution performance by promoting desupersaturation. This study adds to the increasing evidence that residual crystallinity can have a minimal impact on the non-sink dissolution testing of ASDs. However, the level of residual crystallinity allowed must be determined for each formulation, and it depends on the drug's recrystallization tendencies and the polymer's ability to stabilize supersaturation.

The ability to maintain supersaturation during dissolution testing immediately after processing is only one part of evaluating the impact of residual crystallinity on a formulation. It is equally important that a formulation's dissolution profile does not change after stability studies. Changes in a formulation's dissolution profile (e.g., recrystallization during storage) can change the rate and extent of drug absorption (Amidon et al., 1995), which ultimately affects bioavailability. In non-sink dissolution studies, the crystallinity of an ASD can act as a seed for crystal growth or secondary nucleation. This decreases the amorphous solubility advantage and the inability to maintain supersaturation, respectively.

After stability, the crystallinity of Samples 59–4 and 59–5 (stored at 25 °C/60% RH in the closed condition for four months) changed from 0.9% to 1.0% and 0.1% to 0.2%, respectively (see Table 3). This small increase in crystallinity after stability did not decrease the release or promote desupersaturation during dissolution testing. Therefore, copovidone effectively inhibited the growth of crystal seeds (i.e., the residual crystallinity of LY3009120) after processing and after the stability study. This supports the finding that residual crystallinity in formulations 59–4 and 59–5 had a minimal impact during non-sink dissolution testing.

### 4.3 In vivo studies confirm that residual crystallinity did not prevent bioavailability enhancement

Bioavailability studies were performed to determine whether the presence of residual crystallinity affected bioavailability enhancement. At the conclusion of the stability study, Samples 59–4 and 59–5 (stored at 25 °C/60% RH in the closed condition for four months) were selected to understand the impact of residual crystallinity on bioavailability. The samples exhibited a 54.9-fold and 61.9-fold increase in bioavailability, respectively, despite the presence of residual crystallinity. Furthermore, to represent a worst-case scenario, we selected Sample 57–1, which exhibited desupersaturation during dissolution testing. All formulations, despite the presence of residual crystallinity, demonstrated a statistically significant (*p*-value < .045) improvement in bioavailability enhancement compared to the physical mixture (see Table 4). In combination with the dissolution and solid-state characterization, this in vivo study highlights the ability to formulate an ASD with residual crystallinity that (a) does not recrystallize during storage, (b) does not change in dissolution performance over time, and (c) achieves statistically significant bioavailability enhancement compared to the physical mixture.

However, despite Sample 57–1 exhibiting desupersaturation after pH transition, we found no statistically significant difference in AUC_0-Last_ between Sample 57–1 (342 ng·h/mL) and Samples 59–4 (428 ng·h/mL, *p*-value = .3) and 59–5 (483 ng·h/mL, *p*-value = .15), respectively (see Table 4). Desupersaturation occurred during dissolution testing, but Sample 57–1 still achieved a 10-fold increase in solubility compared to the physical mixture, while Samples 59–4 and 59–5 achieved a 15-fold increase in solubility. Therefore, though desupersaturation occurred during dissolution testing, Sample 57–1 was still able to provide solubility enhancement, and it ultimately achieved a 43.9-fold increase in bioavailability compared to the physical mixture during the in vivo study. The high variability in the AUC_0-Last_ values, represented by the percentage coefficient of variation (CV) (see Table 4), hinders the differentiation of the statistical significance of bioavailability enhancement between Samples 57–1, 59–4, and 59–5, at the current sample size. Future studies will include a larger sample size to increase their power.

Additionally, although not statistically significant, Table 4 shows a trend between the F-value (i.e., bioavailability enhancement) and the amorphous fraction of the drug (i.e., the percentage crystallinity of the total drug). Table 3 shows that as the amorphous fraction of LY3009120 decreases, a proportional decrease in the F-value occurs. For example, Sample 59–4 contained 10% less amorphous drug than Sample 59–5, and it showed an 11.3% decrease in F-value compared to 59–5. Similarly, Sample 57–1 had 26.2% less amorphous drug than 59–5 and showed a 29.1% decrease in F-value compared to 59–5. This trend suggests that the amorphous fraction of the drug influences the degree of bioavailability enhancement. Therefore, if a formulation has a decreased amorphous fraction, an increased dose can be used to overcome the decrease in bioavailability.

### 4.4 The balance between crystallinity and degradation in ASDs

This study demonstrates that the presence of residual crystallinity in ASDs may not always be unfavorable, specifically in circumstances in which allowing trace amounts of residual crystallinity enables the level of degradants to fall within an acceptable range without sacrificing the product's long-term stability. As seen in our study, there is an inversely proportional relationship between crystallinity and degradation: As crystallinity decreases, degradation increases (see Table 2). For example, as the LY3009120 formulations approached an entirely amorphous composition, the degradants increased, thus potentially introducing excessive degradation levels in pursuit of eliminating crystallinity. For ASDs, particularly thermally sensitive drugs like LY3009120, lowering the processing temperature or processing time can reduce degradation at the expense of increased crystallinity (Thompson and Williams, 2021). This emphasizes the balance between crystallinity and degradant levels in an ASD.

The performance findings (i.e., stability, dissolution, and in vivo results) emphasize only one aspect during formulation selection. These performance metrics must be coupled with the drug's degradant levels to make a holistic decision during formulation development (Peng et al., 2018). We demonstrate this holistic decision-making in our study by comparing the degradation present in formulations 59–4 and 59–5: Sample 59–4 contains 1.97% total degradants and 0.68% of Degradant A, while Sample 59–5 contains 3.12% total degradants and 1.12% of Degradant A.

However, although Sample 59–4 had higher residual crystallinity levels than 59–5 (i.e., 0.9% vs. 0.1%), Sample 59–4 exhibited similar stability, dissolution, and in vivo performance. This suggests that 59–4 is the preferred formulation due to lower degradant levels, despite the increased amount of residual crystallinity. If degradant levels alone were considered, formulation 57–1 would appear to be the preferred formulation. However, the increased residual crystallinity promotes desupersaturation during dissolution testing and increases the potential for recrystallization during storage; therefore, this formulation's residual crystallinity puts Sample 57–1 at increased risk during development, despite decreased degradant levels.

Therefore, when pursuing a formulation with trace amounts of residual crystallinity, both the performance and the degradant level must be considered. Preferably, and specifically for performance, trace amounts of residual crystallinity do not promote recrystallization during dissolution testing, and the formulation experiences only a marginal loss in solubility. Preferably, for formulation stability, the trace amounts of residual crystallinity would not promote recrystallization during storage. Formulations with high glass transition temperatures, drug loading within the polymer's miscibility limits, and strong drug–polymer interactions are preferable to minimize the risk of recrystallization. It is preferred that there is only minimal generation of degradants. In a situation in which increased degradants are generated, less aggressive processing conditions can be used to minimize degradation at the expense of introducing residual crystallinity. This approach is highly dependent on multiple formulation parameters, but it will help expand the formulation space for some of the most challenging drug formulations, thus it warrants further investigation.

## 5 Conclusion

KinetiSol processing conditions were selected to produce inversely proportional levels of degradation and crystallinity to explore the impact of residual crystallinity during stability, dissolution testing, and in vivo studies. Formulations that contain trace levels of residual crystallinity can prevent further crystal growth during stability, provide solubility enhancement during dissolution testing, and significantly enhancing bioavailability. These facts show that entirely amorphous compositions may not always be required when developing ASDs. This research begins to explore the balance between allowing residual crystallinity and decreasing degradation when an entirely amorphous formulation produces unacceptable degradant levels due to excessive energy requirements. In certain circumstances, the ability to permit trace amounts of residual crystallinity allows for acceptable levels of degradant products while retaining the benefits of a primarily amorphous formulation.

## Declaration of Competing Interest

DAD and ROW are co-inventors on IP related to this paper. The University of Texas Systems has licensed this IP to DisperSol Technologies, LLC. ROW acknowledges an unrestricted educational grant from DisperSol Technologies, LLC to support graduate education.

## Appendix A Supplementary data

Supplementary material Image 1
