# Supplementary material for: Formulating a heat- and shear-labile drug in an amorphous solid dispersion: Balancing drug degradation and crystallinity
Source: Int J Pharm X. 2021 Jul 17;3:100092. doi: 10.1016/j.ijpx.2021.100092 (PMC8683684; doi:10.1016/j.ijpx.2021.100092)
Supplement: Supplementary file 1 — Supplementary material [file mmc1.docx]

**Supplemenatry Figures:**

**
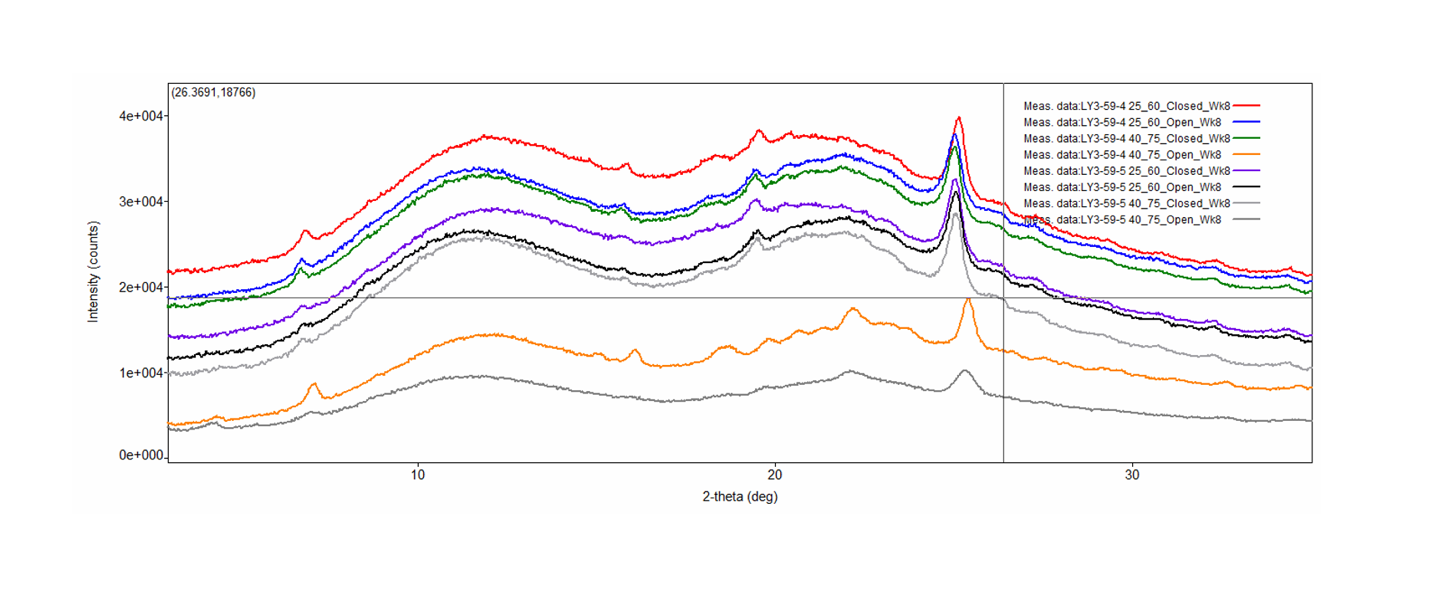
**

**Figure S1: pXRD Overaly of formulations 59-4 and 59-5 at the 8 week stability time point. The overlay demonstrates the lack of sensitivity to differentiate between the low levels of residual crystallinity.**

**
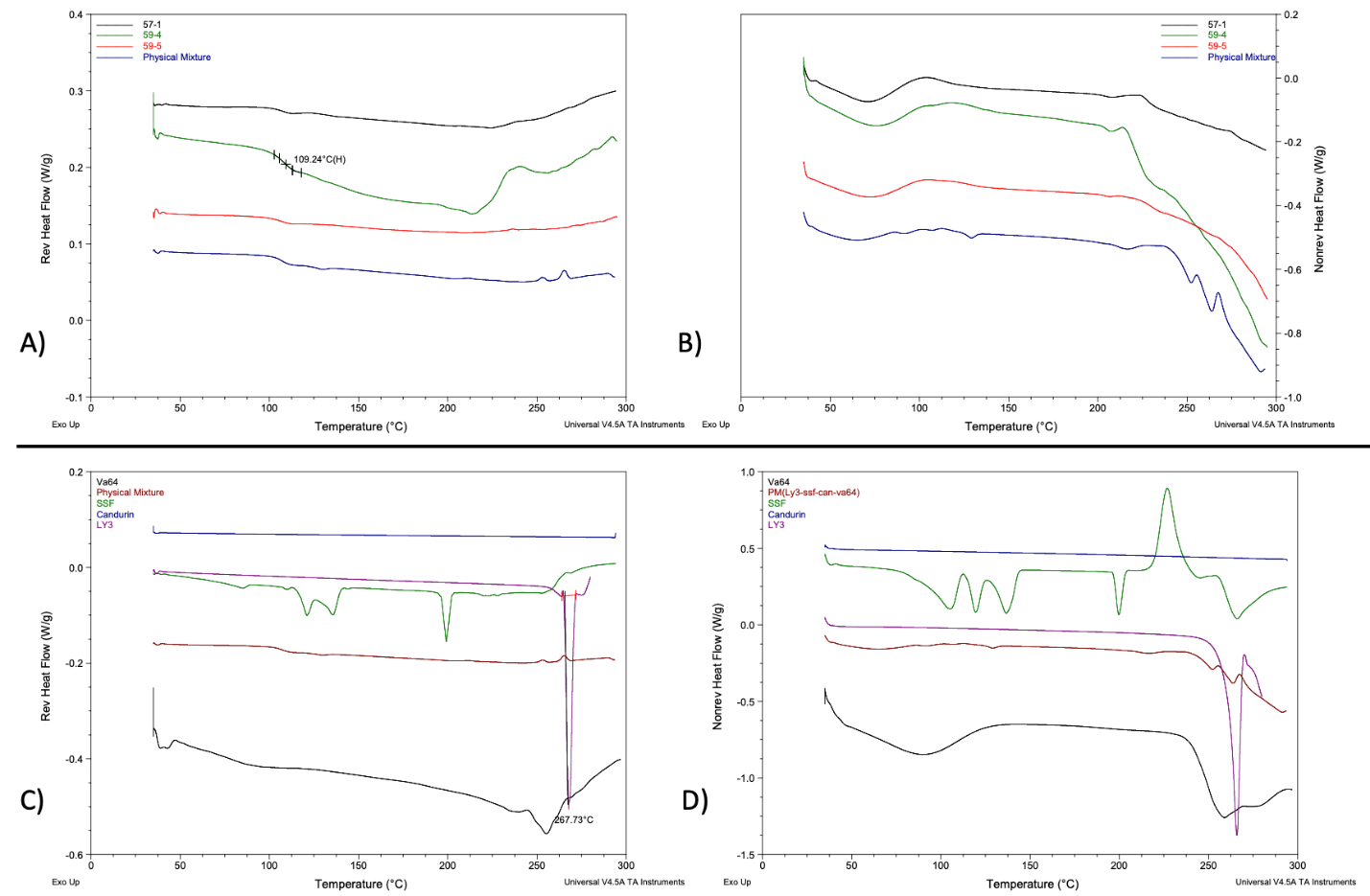
**

**Figure S2: Reversible and nonreversible heat flow overlay of processed formulations (Top) and individual components (Bottom). Figure A identifies the glass transition temperature for formulation 59-4. Figure B) illustrates the polymer degradation that occurs in the region of LY3009120’s melting point. Figure C) identifies the melting point of LY3009120. Figure D) illustrates the occurrence of pure Va64 degradation.**
